# Neural Correlates of Emotion Regulation in the Ventral Prefrontal Cortex and the Encoding of Subjective Value and Economic Utility

**DOI:** 10.3389/fpsyt.2014.00123

**Published:** 2014-09-15

**Authors:** Roberto Viviani

**Affiliations:** ^1^Institute of Psychology, University of Innsbruck, Innsbruck, Austria; ^2^Department of Psychiatry and Psychotherapy III, University of Ulm, Ulm, Germany

**Keywords:** emotion regulation, orbitofrontal cortex, ventromedial prefrontal cortex, subjective value, utility, neuroeconomics

## Abstract

In many studies of the interaction between cognitive control and emotion, the orbitofrontal cortex/ventromedial prefrontal cortex (mOFC/vmPFC) has been associated with an inhibitory function on limbic areas activated by emotionally arousing stimuli, such as the amygdala. This has led to the hypothesis of an inhibitory or regulatory role of mOFC/vmPFC. In studies of cognition and executive function, however, this area is deactivated by focused effort, raising the issue of the nature of the putative regulatory process associated with mOFC/vmPFC. This issue is here revisited in light of findings in the neuroeconomics field demonstrating the importance of mOFC/vmPFC to encoding the subjective value of stimuli or their economic utility. Many studies show that mOFC/vmPFC activity may affect response by activating personal preferences, instead of resorting to effortful control mechanisms typically associated with emotion regulation. Based on these findings, I argue that a simple automatic/controlled dichotomy is insufficient to describe the data on emotion and control of response adequately. Instead, I argue that the notion of subjective value from neuroeconomics studies and the notion of attentional orienting may play key roles in integrating emotion and cognition. mOFC/vmPFC may work together with the inferior parietal lobe, the cortical region associated with attentional orienting, to convey information about motivational priorities and facilitate processing of inputs that are behaviorally relevant. I also suggest that the dominant mode of function of this ventral network may be a distinct type of process with intermediate properties between the automatic and the controlled, and which may co-operate with effortful control processes in order to steer response.

## Introduction

Studies of cognition, especially those concerned with selective attention or cognitive control, have exerted a profound influence on models of emotion regulation in functional neuroimaging (fMRI). Cognitive approaches originally viewed attention as a means of protecting the cognitive apparatus from flooding from external stimuli, an aim achieved by interposing a bottleneck or filter on information flow (1). Even if revised to accommodate a rich set of empirical findings, subsequent models of attention largely inherited the contrast between the top-down regulatory role of attention on the one hand, and stimuli attempting to influence cognition from the bottom-up on the other. These models include those based on the influential idea of attention as a limited resource process (2), as well as contemporary approaches that merge attention and executive processes within a comprehensive theory of cognitive control based on attentional allocation mechanisms (3–5).

KEY CONCEPT 1. Cognitive control An encompassing term for control processes defined by features of executive functions (such as being based on limited resources or being subject to interference). Modern research on executive function emphasizes its relationship with working memory and endogenous attentional mechanisms. Another term associated with cognitive control is “top-down,” as opposed to “bottom-up” content attempting to gain access to working memory. In contrast, “control” (without the qualification “cognitive”) is used here more generically to denote any kind of influence or regulation.

KEY CONCEPT 2. Emotion regulation The process that steers generation of response in the presence of emotional stimuli. Most theories of emotion stress the capacity of emotional stimuli to grab attention or engender action tendencies (for example, approach or avoidance after emotional stimuli of positive or negative tone). Emotion regulation refers to mechanism that correct or influence this direct response to emotion. The dual-process model of emotion regulation emphasizes the role of cognitive control to implement this correction.

**Figure 1:**
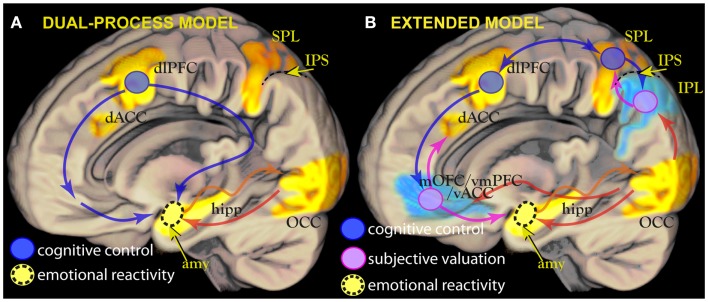
**(A)** Left: the dual-process model posits the existence of two interacting processes: sensory encoding of emotionally salient stimuli, principally in the amygdala (amy, in yellow), and cognitive control centers in the prefrontal cortex such as the dorsolateral prefrontal cortex (dlPFC, blue circle). In this model, cognitive control centers inhibit or constrain the activity in the amygdala elicited by emotionally arousing stimuli. Amygdalar activity contributes to the salience of stimuli by influencing visual cortical regions directly [yellow arrow, see Ref. (13) for details]. **(B)** Right: a revised model in which subjective valuation areas such as the ventral cingulus/orbitofrontal cortex (mOFC/vmPFC/vACC) or the inferior parietal lobule (IPL, pink circles), usually deactivated during focussed tasks (in blue), contribute to determining response together or in alternative to control centers in the dorsal attentional network (blue circles). This contribution is revealed by modulation of deactivations or brief “flashes” of activity when attention is refocused (35). In the revised model, mOFC/vmPFC provides information about motivational priorities of stimuli identified in parietal association areas. This information is computed by integrating primary motivational states with representations of value and/or contingency.

Largely inspired by these models, fMRI studies have demonstrated the dissociation between the neural substrates associated with cognitive control and those associated with the perception of emotionally salient stimuli. Working memory and attentional tasks of executive nature activate a dorsal network centered on the dorsolateral and dorsomedial prefrontal cortex [dlPFC, dACC; (5–9)]. In contrast, emotionally arousing stimuli activate the amygdala, a gray matter structure on the medial face of the temporal lobe (10–13). Emotional stimuli appear to enjoy preferential processing (13–16), presumably because of the importance of the information they convey on the environment (17, 18). However, this also means that they constitute a challenge for attentional control processes when emotion is a source of interference, as shown in studies where emotional stimuli used as distractors and executive control are pitted against each other [for reviews, see Ref. (19–21)]. This distinctive challenge is the hallmark of cognitive control-based models of emotion regulation mechanisms (22–25). According to these models, the prefrontal areas associated with cognitive control down-regulate activity in the limbic system (Figure 1A). These models have been applied to data about individual differences in emotion regulation styles (26–30), their possible alteration in pathology (31, 32), and data about changes during therapy (33, 34). In the following, this psychobiological model will be referred to as the “dual-process model” of emotion regulation.

KEY CONCEPT 3. Salience The capacity of a stimulus to grab attention or, especially in settings where attentional effects in laboratory animals are not directly observable, to be influential in determining response. This term is often qualified by the quality of the stimulus that is thought to be responsible for the attention-grabbing effect. Sensory salience, for example, refers to the perceptual intensity of stimuli, such as a loud noise, which makes them likely to be attended. In the behavioral literature of choice, incentive salience refers to the capacity of representations of reinforces to motivate response.

KEY CONCEPT 4. Dual-process model Any model that employs two process types in a psychological explanation. Often, but not necessarily, these two processes refer to some version of the top-down and bottom-up distinction. In the present review, a dual-process model of emotion regulation is considered.

However, there are also reasons to be increasingly critical about this research program. One reason is that while the empirical evidence on the regulatory effect of cognitive control on emotion is extensive, much less attention has been given to the question of how emotion may be regulated through other mechanisms. Furthermore, the unbalance between cognitive control processes on the one hand and the tendency of emotional information to break through control on the other appears to be present in most affective disorders, implying the lack of discriminatory capacity of the model. This is a problem for attempts to use clinical neurosciences in a clinical context to refine diagnosis and inform choice of treatment (36–38). Another reason is that not all findings on the selection of emotional information to generate response appear to be attributable to networks associated with cognitive control or to be classifiable within a simple dichotomy (39–41).

This focused review will examine questions raised by neuroimaging findings and theories on the function of the medial orbitofrontal and the ventromedial prefrontal cortex (mOFC/vmPFC) for the cognitive control of emotion. These questions emerged in the original study that occasioned this focused review (42), but are present in many fMRI studies concerned with emotion and its regulation. In the study by Benelli et al. (42), we exposed participants in the scanner to different versions of short texts describing what happens in scenes that may be interpreted as referring to emotional issues such as loss, illness, or discord. The different versions of the texts, the same in all participants, had the same content, but varied in the amount of emotional or abstract terms. After the scan, participants were asked to recount what happened in the scenes in writing. We then looked at individual differences in the use of emotional terms in these accounts, and regressed them on the changes in brain activation while they were reading the text in the scanner. We considered this to be a form of emotion regulation, as it was emotional material that was being selectively left out. Strikingly, we found that the use of emotional words did not correlate with changes in areas associated with cognitive control, such as dlPFC. Instead, it was significantly associated with modulations of deactivations during the reading task, including the mOFC/vmPFC region (BA32). We interpreted this finding as evidence that these areas influenced the tendency to ignore or encode specific aspects of the texts.

This interpretation, however, left open the issue of a precise characterization of the mechanisms through which material was taken in or ignored, and of why this took place in the context of a deactivation of the mOFC/vmPFC area. Also, open is the issue of the relationship of these mechanisms with cognitive control and its neural substrates. This is an important issue given the enormous theoretical importance of the notion of cognitive control for the theory of emotion regulation (Figure 1A). If there is evidence for a form of regulation based on mOFC/vmPFC, does it look like the top-down control associated with activation of dorsal attentional areas? If not, what is the role of mOFC/vmPFC in the attentional architecture of the mind? In this review, I will argue that mOFC/vmPFC may work together with the inferior parietal lobule (IPL) to influence attentional processes on the base of emotional information and that this mechanism may underlay the form of control attributed to this region.

### mOFC/vmPFC and control

In many tasks considering the effects of attentional instructions, activation of the mOFC/vmPFC has been interpreted as the neural correlate of a form of control specifically directed to emotional content (43–45), or as the neural correlate of the inhibition of areas involved in the detection of emotionally arousing stimuli such as the amygdala (46–50). Importantly, the mOFC/vmPFC is also specifically involved in reversal learning and the extinction of aversively conditioned stimuli (51–55). Structural and functional indices of connectivity between mOFC/vmPFC and the amygdala are also reported to be associated with genetic determinants of vulnerability to affective disorders [(56–59); see, however, Ref. (60, 61)]. Furthermore, the disruptive and disinhibitory effects of lesions of mOFC/vmPFC on behavior have long been known (62).

However, several findings mark the difference between mOFC/vmPFC and more dorsal areas associated with cognitive control. Extensive neuropsychological evidence shows that deficits following OFC damage dissociate from those that follow from damage of dorsal prefrontal areas (52, 63–66). Unlike the dorsal network of which dlPFC is part, mOFC/vmPFC is deactivated by focused, effortful tasks (67–70). This feature is shared by a number of areas referred to as the default network system (71), a set of interconnected regions deactivated by executive tasks but showing higher activity at rest [hence, the deactivation of mOFC/vmPFC reported by Benelli et al. (42) was typical; the originality of the finding concerned the association with the later tendency to recount the emotional attributes of the scene].

Another problematic finding for the emotion regulation view of mOFC/vmPFC is its activation in tasks where no control on emotional material is required (72–74). Furthermore, two independent studies specifically designed to compare recruitment of prefrontal areas when facing neutral and emotional distracters found that the same prefrontal dorsal areas were responsible for both (75, 76). Also, comparisons of cognitive distraction and emotion regulation tasks show considerable overlap of prefrontal activation in the dorsal areas (77, 78). These data speak against the view of mOFC/vmPFC as a *cognitive* control area specialized for emotional content [see also, Ref. (39, 55, 61, 79)].

In summary, there is ample evidence for a regulatory involvement of mOFC/vmPFC in the generation of response when emotion plays a role. However, it is difficult to conclude that the regulatory function of this area is of executive nature. The issue is then what kind of control function may be associated with mOFC/vmPFC, especially if considered with respect to the attentional architecture of the mind that defines the notion of cognitive control associated with dlPFC and other areas mapped to executive processes.

### Neural correlates of the encoding of subjective value and economic utility in mOFC/vmPFC

Interestingly, activation of mOFC/vmPFC has been reliably detected in studies that were apparently unconcerned with issues of emotional control, and were conducted by researchers active in the field of neuroeconomics. These studies have shown that mOFC/vmPFC is associated with the computation of the subjective value of stimuli or outcomes (80–87). This computation may correspond to evaluating an empirical version of “marginal utility,” i.e., the criterion by which choices are made according to the desirability or aversiveness of objects or action outcomes while considering internal indicators of needs or satiety (81, 86). These areas are thought to encode learned representations of reward and punishment and/or their reversal (80, 88). They are, therefore, crucial to understand internal representations acquired during long-term interactions with the outside world, and the role of these representations in determining response in relation to the current or long-term motivational setup of the individual.

KEY CONCEPT 5. Neuroeconomics An emerging field that studies the psychobiological mechanisms of choice and economic behavior. This field has important antecedents in earlier research fields such as mathematical psychology, learning theory, and psychophysics.

KEY CONCEPT 6. Subjective value In neuroeconomics, the criterion of desirability on the basis of which a choice is made. A related or equivalent term is “preference.” The notion of subjective value is the neuroeconomic equivalent of “utility” in economic theory. Economic utility differs from subjective value because it is considered an unobservable entity, inferred indirectly from an axiomatic theory of choice. The present review considers the possibility that preferences may shape response to emotional stimuli without resorting to cognitive control.

The neuroeconomic perspective on the orbitofrontal cortex is consistent with previous studies on the anatomical organization and function of this region. The OFC is connected with sensory cortical areas of all modalities [for review, see Ref. (89, 90)]. Studies in laboratory animals have shown this cortex to be activated by the identity of rewards or aversive stimuli, irrespective of their spatial location or sensory features [for reviews, see Ref. (79, 91)].

For the theory of emotion regulation, the crucial issue is whether “utility” or its neural counterpart “subjective value” may override stimulus salience in the generation of response without recourse to effortful mechanisms associated with cognitive control. Two examples are resisting the impulse to acquire a smaller sum immediately rather than a larger sum later (92), and the selection of optimistic thoughts in the presence of salient negative stimuli (60).

Kable and Glimcher (92) tested the hypothesis that individuals who resisted the impulse to cash in a smaller sum immediately instead of a larger sum at a later point in time would recruit cognitive control areas (93). This hypothesis was not confirmed by the data, which showed subjective valuation areas such as the mOFC/vmPFC to be directly involved in computing the desirability of choosing the immediate or delayed option, without recruiting dorsal areas associated with cognitive control. Note that in this situation, as well as in more prototypical emotion regulation paradigms, cognitive control and the related dorsal prefrontal areas may be recruited in appropriate circumstances to increase selection of the more “controlled” option (94–96), as predicted by the model that attributes inhibition of impulse to executive processes. This is not surprising in view of the data mentioned above, which show recruitment of the same dorsal prefrontal areas when controlling emotional and non-emotional distractors (75, 76). The key finding, however, is that in other circumstances these areas may not be recruited, suggesting that the form of control mapped to dorsal attentional areas may not be the only determinant of “controlled” choice, and that different strategies may be available to produce similar responses.

The study by Viviani et al. (60) is a perfusion imaging study in which participants were asked to assemble one of two possible sentences from a set of scrambled words. This task uncovers the propensity to favor positive thoughts when the alternative sentences have an emotional connotation (for example, the set “is bleak the future bright” can be reassembled into either “the future is bright” or “the future is bleak”). Healthy individuals avoid the negative alternative, forming only 20–30% negative sentences. Individual variability in this propensity correlates with depressiveness, and in certain circumstances is predictive of relapse in remitted depressives (97). This task originated within a model positing that cognitive control processes are responsible for the avoidance of the negative alternative in individuals with vulnerability to depression (98). Because negative words are generally more salient than positive words, the dual-process model predicts that control processes be recruited to exclude negative words in order to achieve a desired mental state (99). In contrast to the prediction of this model, the vmPFC and other ventral prefrontal and parietal areas were found in this study to be more active when producing spontaneous sentences, while dorsal prefrontal areas were less active. Furthermore, the number of sentences used, but not the propensity to avoid the negative alternative, was associated with individual differences in working memory capacity. Dorsal prefrontal areas, however, were active if avoidance of negative sentences followed an explicit instruction of the experimenter.

A possible explanation of the findings of these two studies is that response may be influenced by preferences represented in ventral areas such as OFC and vmPFC, which may override the salience of emotionally arousing stimuli or the impulse to collect an immediate reward directly instead of requiring the intervention of cognitive control. In the example on the choice of sentences, this means that we choose “the future is bright” more often because we prefer this thought, not because “bright” is more salient than “bleak.” While perhaps intuitively plausible, this possible explanation raises questions on the organization of the mind that makes this form of regulation of response possible. The dual-process model of emotion regulation is grounded in a solid theoretical and empirical framework that documents the importance of cognitive control in the cognitive architecture of the mind. To move in this direction, a characterization of the kind of process that may be responsible for regulating response to emotional stimuli through the expression of preferences is required.

## Are the Encoding of Subjective Value or Other Processes Mapped to mOFC/vmPFC Automatic?

Particularly relevant to evaluating the possible role of mOFC/vmPFC as the neural correlate of a control process are claims in the decision-making literature that the valuation signal detected in this cortical region is computed automatically, and is detectable even in the absence of choice tasks (100, 101). Rushworth et al. (85) marshal data that may not be consistent with this view. They note that the signal in mOFC/vmPFC is not observed in all studies where subjective value may be computed. However, one rarely finds that all defining features of automaticity are satisfied simultaneously (102); obligatory processing, in particular, is seldom absolute, nor is the absence of interactions with attentional processes (103–105). The signal in the amygdala observed in concomitance with emotionally arousing stimuli, for example, is widely considered to arise pre-attentively (13, 106, 107), but is nevertheless modulated by the task set (108, 109).

KEY CONCEPT 7. Automatic In the present review, the term automaticity refers to processes evoked by the stimulus and running without monitoring, whose initiation is not necessarily deliberate, and that are not subject to strong capacity limitations. The perceptual encoding of emotional stimuli, including their arousal properties, is an example of an automatic process. However, the literature contains several different approaches to the definition of automaticity, including notions such as emerging from repeated practice, or of running largely beyond subject control. A related characterization of an automatic process, referring to the flow of information, is “bottom-up,” as opposed to “top-down.”

The mOFC/vmPFC value signal has other characteristics that suggest its association with a complex computation, rather than a simple assessment of the properties of the stimulus as may be expected by an automatic perceptual process. In some studies, for example, the signal reflected the difference in value of the chosen and the unchosen options (110, 111), or the discount due to delays in obtaining a reward (92, 94). While the complexity of the computations attributed to a process does not suffice in itself to classify it as effortful or resource-limited, it does suggest that it may be influenced by several factors.

An interesting perspective on this issue is given by the notion of appraisal from appraisal theories of emotion (112–114). In these theories, appraisal is the assessment of the significance of the environment and of interactions with the environment for the goals and concerns of the individual, i.e., for everything one cares about. This process is thought of as setting the value of one or more “appraisal variables” that categorize aspects of the environment that carry information about one's goals and concerns. The valence of an emotional episode depends on the outcome of this computation.

There are obvious parallelisms between the notions of appraisal and computation of subjective value [it has also been noted that the data collected from mOFC/vmPFC in neuroimaging studies of affect are consistent with a role as a “generator of affective meaning”; see Ref. (115)]. Notwithstanding its complexity, the appraisal process has been shown by emotion researchers to present many features of automaticity (116) or lack of intentionality [in the sense of not being deliberate (117)]. This strongly suggests that, even if not completely automatic, value-setting operations in human beings do not require controlled processes.

## How Does Information on Value Affect Response?

Studies of reinforcement in rodents have shown that information on potential rewards influences behavior through the interaction of two dissociable processes, one controlling habitual and the other goal-directed actions (80, 118). Habitual actions, or simply “habits,” are those that, as in classic theories of reinforcement, link a stimulus to a response. Once established, responses are triggered reflexively by the appearance of the stimulus. Habits emerge by overlearning stimulus-response associations, and may therefore present all features of automaticity that are shown to follow from repeated practice (119). Goal-directed actions, in contrast, are undertaken using information about what their outcome would be, and about the current utility of this outcome. This is accomplished through representations of the causal dependency of action and outcome (referred to as “contingencies”) and the utility of the outcome (“incentive value”). These representations allow goal-directed actions to be flexibly modulated by changes or reversals of contingencies between action and outcomes and by changes in the utility of the outcome. Importantly, representations of utilities are in turn modulated by motivational factors shaped by the previous experience with the environment (120). Another type of mechanisms affecting response involves the evaluation of cues. Cues associated with specific rewards may motivate the choice of the course of action leading to the reward evoked by the cue (80, 121).

Studies of reinforcement implicate mOFC/vmPFC in the representation of both incentive value and contingencies (121–123) and in the use of cues to select response (121, 124). In human beings, fMRI studies of reinforcement have confirmed the involvement of mOFC/vmPFC in the representation of outcome values in goal-directed action [for reviews, see Ref. (81, 85, 123)] and of contingencies (125–127). Furthermore, data in non-human primates and in human beings demonstrate the modulation of mOFC/vmPFC by primary motivational states (128–130).

Within this framework, the representation of contingencies in mOFC/vmPFC provides an explanation of the involvement of this structure in reversal learning. Characterized as inhibition in studies adopting dual-process models of emotion regulation, reversal learning involves updating contingencies stored in mOFC/vmPFC [see Ref. (131) and the discussion in Ref. (121)]. Likewise, differences in discounting delayed rewards (92) may be implemented by modulating mOFC/vmPFC representations of value. Hence, the flexibility of goal-directed processes and their role in determining behavioral choices may explain the finding of an association of behavioral control with mOFC/vmPFC function, especially when emotion or rewards are involved.

A distinctive quality of human mOFC/vmPFC is its activation in fMRI studies in which rewarding or aversive outcomes play no nominal role (132–134). In a review of these data, Elliott et al. (132) have shown how these activations are elicited in tasks involving feedback or guesswork, where selection of stimuli on the basis of familiarity or responses on the basis of a feeling of “rightness” may constitute generalizations of reward value. They were also able to show the dissociation of response between mOFC and dlPFC in studies where guesswork and difficulty of the task related to the instruction were varied independently (135). This model may explain the ventral/dorsal dissociation of substrates in participants choosing sentences spontaneously or following an instruction in the study by Viviani et al. (60). More recent models of the computational role of mOFC/vmPFC see it as tracking outcome expectancies especially in situations in which these outcomes are uncertain, or when information about outcomes needs to be disambiguated using internally stored information (136, 137).

The flexibility afforded by representations of outcomes and contingencies has motivated many researchers of reinforcement to refer to goal-directed processes as executive, in contrast with the automatic quality of habits. However, several arguments may be formulated against identifying goal-directed processes with executive control. One is that goal-directed processes are sensitive to motivational factors (120). In contrast, executive function may be recruited to steer response in disregard of motivation. Situations in which motivation and executive control dissociate may be difficult to devise in experimental settings such as reinforcement in rodents, but are common in emotion regulation research, where they are an important reason to associate executive function with emotion regulation. It is not clear that the flexibility of goal-directed processes observed in rodents may provide a good model of executive function in human beings, since choices based on motivational factors may be more appropriately characterized as following one's inclination or preferences rather than exerting effortful control. As we have seen, there is ample evidence that human appraisal of value is largely automatic (116). Another group of arguments is based on data, already mentioned above briefly, showing that lesions of the human orbitofrontal cortex affect decision taking, but leave working memory intact (65, 138, 139). Furthermore, fMRI studies associate working memory with dorsal parts of the prefrontal cortex, not with mOFC/vmPFC (6). These arguments suggest that goal-directed action as characterized by studies of reinforcement may be neither fully automatic nor executive, like encoding of subjective value in mOFC/vmPFC, even if it contributes to steering response.

## Subjective Value as a Factor in Attentional Orienting

If representations of utilities in mOFC/vmPFC can influence response without resorting to effortful executive processes, how is this accomplished in terms of an attentional account? In studies of non-human primates where choice is mapped to saccadic movements coded in the frontoparietal attentional network, activity in the parietal map mirrors activity in the mOFC/vmPFC where subjective value is computed (86). Hence, an influential neuroeconomic model treats mOFC/vmPFC and parietal areas as a unitary network for the computation of choice, suggesting that functional connectivity between mOFC/vmPFC and attentional areas in the parietal cortex must convey information on utilities (88, 140).

Based on these data, it is straightforward to formulate a mechanism of influence of utility representations on attention in terms of the biased competition model. In this model, top-down control exerts its influence by biasing the competition between incoming stimuli vying for inclusion into working memory (141) or between activated input–output associations (142). In the absence of top-down control, the outcome of this competition is determined by stimulus salience (such as sensory salience). To account for the influence of utility representations, this model can be extended by having mOFC/vmPFC provide additional sources of salience related to utility [such as representations of potential “incentive salience” of stimuli or outcomes (120, 143)]. As a result, it may be possible to override sensory salience by following one's motivational bias without invoking executive processes. These latter may override both sensory and incentive salience in some cases, and bolster up incentive salience in others. In this extended model, however, executive function and motivational biases are distinct sources of control.

There is direct support for this model in human studies showing the modulation of attention by orienting to previously rewarded targets or distractors [reviewed by Anderson (144); see also Ref. (145)]. Importantly, these researchers were able to show the existence of attentional capture by rewarded distractors independently from sensory salience of stimuli and top-down bias [“value-driven attentional capture” (146, 147)].

An important insight emerging from these studies is that the influence of utility representations on cognition may take place through attentional orienting. Modern characterizations of attention see it as resulting from the interaction of distinct processes (35). A first process, located in dorsal attentional areas (frontal eye fields and superior parietal lobule), is responsible for maintaining the focus of attention. A second process, mapped to ventral areas (the inferior parietal lobule and adjacent temporal cortex, IPL), provides information of “behaviorally relevant” changes to promote reorienting to stimuli shat should receive high priority in complex environments (148–150). Orienting processes associated with ventral parietal areas are stimulus-bound, i.e., respond to external stimuli. However, this response differs from the automatic attending of sensory salient stimuli, as IPL responds to “behaviorally relevant” stimuli even when less sensory salient. At the same time, they also react to stimuli that correspond to the task set (35), thus presenting features that are neither automatic nor executive, or a mixture of both [for a detailed discussion, see Ref. (40)].

There are three reasons to look closely at studies of spatial orienting. The first is that they point to a specific type of cognitive process, exemplified by orienting, to handle information on “behavioral relevance,” which I suggest is identical to the already wide class of information about value and contingency mapped onto mOFC/vmPFC. The second is that they point to IPL as a region where this information is integrated with information about the sensory identity of stimuli during attentional processing, in agreement with neuroeconomic models of choice based on primate data (88, 140). The third is that they describe the behavior of the signal from inferior parietal areas in neuroimaging studies as characterized by functional modulations of deactivations (35), a behavior, which may apply to mOFC/vmPFC too. This is only one of several homologies between IPL and mOFC/vmPFC (Table 1), suggesting that these two areas may be more similar than previously thought. If IPL and mOFC/vmPFC work together to integrate information about utilities with attentional processes, their relative activation level may be associated with the extent to which incentive bias is allowed to influence competition between stimulus representations, and their deactivation the extent to which top-down bias of executive nature dominates (Figure 1B).

**Table 1:** **Adenosine receptor expression in immune cells and signaling pathway**.

mOFC/vmPFC	Ventral parietal orienting areas
Lesions cause disregard for outcomes of behavior with relatively preserved executive function (138)	Lesions cause attentional deficit (neglect) with relatively preserved executive function (151)
Modulated by subjective value and preference in choice (see main text for references)	Modulated by “behavioral relevance” in spatial attention (35)
Involved in suppressing irrelevant memories, but separately from executive function (152)	Activated by spontaneously evoked memories (153, 154)
Deactivated by focused cognitive tasks, in contrast to dorsal counterpart (67)	Deactivated by focused cognitive tasks, in contrast to dorsal portion (35)
Commonly modulated by emotional material (115, 155, 156)	Often modulated by emotional material [evidence reviewed in Ref. (40)]
Philogenetically evolved as secondary olfactory cortex (157)	Secondary/semantic association areas (158)
Functionally interoperating with ventral parietal areas in neuroeconomic studies of choice (92, 159)	Functionally interoperating with mOFC/vmPFC in neuroeconomic studies of choice (92)
Functionally opposed (155, 156, 160), but interoperating with an adjacent dorsal area (50)	Functionally opposed, but interoperating with an adjacent dorsal area (35, 161)
Not clearly associable with either completely automatic or controlled processes (argument presented here)	Associated with processes with intermediate characteristics between automatic and controlled [evidence reviewed in Ref. (40)]

There are several data suggesting that IPL and mOFC/vmPFC may work together. Extensive connections exist between them (162–164). A comprehensive meta-analysis of neuroimaging studies of the computation of subjective value documented the tendency of parts of mOFC/vmPFC to co-activate IPL [(159); see also Ref. (165)]. Signal from IPL has also been associated with variations in the contingencies of instrumental choices (126, 166). However, several neuroimagers have argued in favor of fractionating IPL into heterogeneous functional areas rather than seeking functional principles unifying multimodal associative and attentional orienting processes.

A few fMRI studies of spatial attention also show activation in mOFC/vmPFC when using stimuli with emotional valence [although not always: (167)]. Two of these studies have shown modulation of signal in the anterior IPL/postcentral gyrus (168, 169) and the OFC [(169); in the study of Fredrikson et al. (168), the OFC was outside the scanned volume]. Small et al. (133) documented a signal in OFC associated with expectations in a spatial attentional task, and characterized it as “motivational” influence on spatial attention. Also, Pourtois et al. (170) found evidence for OFC modulation at the presentation of targets after emotional spatial cues (faces with emotional expression).

## Conclusion

The target paper of this focused review unexpectedly reported that individual differences in the tendency to encode emotional information were associated with significant modulations of areas deactivated by the task, including mOPC/vmPFC and inferior parietal areas. I have argued here that very different sources of evidence may together make sense of this observation, suggesting that these areas may encode a motivational bias influencing attention and response.

The view that I articulated here departs from prevalent models of emotion regulation based on a simple dichotomy between top-down and bottom-up processes. Abandoning the Procrustean bed of dual-process models may introduce unappealing complexities, but may reward us with more accurate accounts of the interaction between motivation and cognition. In the future, these more complex accounts may be important to fully capture the variety of the phenomenology and psychopathology of affect.

## Conflict of Interest Statement

The author declares that the research was conducted in the absence of any commercial or financial relationships that could be construed as a potential conflict of interest.
